# Modified Code Index Modulation Scheme Based on Multi-Carrier M-ary DCSK System

**DOI:** 10.3390/e27030216

**Published:** 2025-02-20

**Authors:** Bin Yu, Guo-Ping Jiang, Hua Yang, Ya-Qiong Jia, Hao Liao, Xin Li

**Affiliations:** 1 School of Computer Science, Nanjing University of Posts and Telecommunications, Nanjing 210023, China; yubin@hnit.edu.cn; 2 School of Electrical Information Engineering, Hunan Institute of Technology, Hengyang 421002, China; jyqhugong@hnit.edu.cn (Y.-Q.J.); 2003001368@hnit.edu.cn (H.L.); 2021001010@hnit.edu.cn (X.L.); 3 School of Automation, Nanjing University of Posts and Telecommunications, Nanjing 210023, China; 4 School of Electronic and Optical Engineering, Nanjing University of Posts and Telecommunications, Nanjing 210023, China; yangh@njupt.edu.cn

**Keywords:** chaotic communication, differential chaos shift keying (DCSK), multi-carrier, code index modulation, M-ary modulation

## Abstract

In this paper, a modified code index modulation scheme based on the multi-carrier M-ary DCSK system, referred to as a MC-MCIM-MDCSK system, is proposed. In the proposed MC-MCIM-MDCSK system, a modified code index modulation scheme is carried out, which selects two orthogonal Walsh codes for realizing the M-ary DCSK encoder. The theoretical BER expression of the proposed system is obtained over additive white Gaussian noise (AWGN) and the multipath Rayleigh fading channels. Abundant simulation results showed that the simulation results matched the theoretical results. The energy efficiency (EE), spectrum efficiency (SE), the data rate, and the complexity of the proposed system were carefully analyzed. The BER performance of the proposed system was compared with those of other systems. It is shown that the proposed system has better BER performance than its competitors.

## 1. Introduction

Chaotic signals are widely used in communication systems due to their initial value sensitivity, wide spectrum, and noise-like characteristics [1,2,3]. Among many chaotic modulation schemes, differential chaos shift keying (DCSK) is the most popular non-coherent one [4]. However, DCSK has certain shortcomings, such as relatively low energy efficiency and data rate [5]. For improving the performance of DCSK system, many enhanced systems have been proposed, such as SR-DCSK [6], NR-DCSK [7], CS-DCSK [8], M-DCSK [9], and so on.

For high data rate and fighting with the poor transmission environment in high data rate mobile communications, a multi-carrier (MC) transmission scheme was utilized in chaos-based communication systems, such as multi-carrier DCSK (MC-DCSK) [10], multi-carrier chaos shift keying (MC-CSK) [11], multi-carrier M-ary differential chaos shift keying (MC-MDCSK) [12], and so on.

Recently, index modulation (IM) has been developed for chaos-based communication systems to improve the data rate and energy efficiency. Code index modulation (CIM) is a type of index modulation that utilizes Walsh-coded indexing to transmit information bits [13]. The optimization scheme of CIM-DCSK was proposed in [14], which combines DCSK with code index modulation. By using noise reduction techniques and optimizing the power coefficients of the reference and information-bearing signals, the performance of the CIM-DCSK system is improved. To obtain high spectral efficiency, high data rate, and excellent performance, a novel M-ary Orthogonal Multilevel Differential Chaos Shift Keying system with Code Index Modulation (CIM-OM-MDCSK) system is proposed [15]. To further boost both SE and EE, a CIM-MDCSK system is proposed [16]. Two Walsh codes are utilized for generating two orthogonal components for information bearing signal in the CIM-MDCSK system. The parallel CIM-DCSK (PT-CIM-DCSK) system is proposed to resist multipath fading and to increase the data rate [17]. For improvements in the date rate and bit error rate (BER) performance, a hybrid DCSK scheme combining cyclic shift keying and code index modulation (CSK-CIM-DCSK) system was proposed in [18]. In this system, the cyclic shift keying modulation is adopted to carry additional information bits. However, these systems cannot simultaneously achieve high data rates, high spectral efficiency, and high energy efficiency.

Inspired by CIM and MC-MDCSK, a modified code index modulation scheme based on the multi-carrier M-ary DCSK (MC-MCIM-MDCSK) system is proposed in this paper. In this system, a modified code index selecting method is adopted. And the M-ary DCSK encoder is realized by chaos signal and two orthogonal Walsh codes, which is different with the implementation method of the M-ary DCSK encoder in CIM-MC-MDCSK [19]. In brief, the main contributions of our work are as follows:

(1) A modified CIM communication system based on MC-MDCSK is proposed, referred to as a MC-MCIM-MDCSK system. This system adopts a new code index selecting method which selects two orthogonal Walsh codes for realizing the M-ary DCSK encoder.

The newly proposed system combines multi-carrier and code indexing techniques. Multiple parallel signals carry information on mutually orthogonal Walsh codes.

(2) The BER formula of the proposed MC- MCIM-MDCSK system is derived over the AWGN and multipath Rayleigh fading channels. The simulation results are in good agreement with the theoretical analysis results. The comparative analysis among the proposed system and the existing systems are shown to confirm the superiority of the proposed system.

(3) The SE, EE, the data rate, and the complexity of the proposed system are analyzed and compared with other systems. The proposed system performs the best in EE, SE, and the data rate when the number of the parallel group is relatively large.

The rest of this paper is as follows. The system model of the MC-MCIM-MDCSK system is shown in Section 2. Theoretical analysis of the BER performance and other performances, such as SE, EE, the data rate, the time complexity, and the memory complexity, are given in Section 3. Section 4 gives the simulation results and discussions. Section 5 shows the conclusion.

## 2. System Model of MC-MCIM-MDCSK

### 2.1. Transmitter

**Figure 1:**
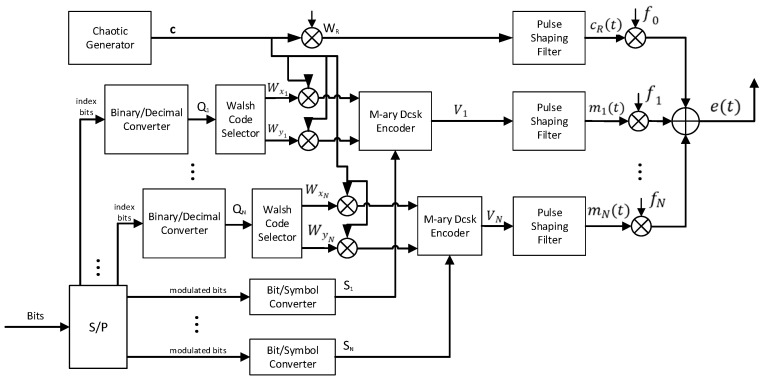
The block diagram of a MC-MCIM-MDCSK transmitter.

Figure 1 shows the block diagram of MC-MCIM-MDCSK transmitter. The information-bearing data is divided into N groups by using a serial-to-parallel converter, and each group data includes $p_{1}$ index bits and $p_{2}$ modulated bits. The chaotic signal $c=[c_{1},c_{2},\cdots c_{\theta }]$ is generated by a second-order Chebyshev polynomial function (i.e., $c_{i+1}=1-\varphi c_{i}^{2}$) with initial conditions $c_{1}=0.9058\;$ and the parameter φ=2. The index bits are converted into the decimal symbol $Q_{n}(n=1,2,\cdots N)$. The modulated bits are converted into the constellation symbol $S_{n}(n=1,2,\cdots N)$. The decimal symbol $Q_{n}$ is used for selecting the paired Walsh codes sequences $W_{x_{n}}$ and $W_{y_{n}}$ which are obtained from the same Hadamard matrix. The indices of the paired Walsh codes sequences $W_{x_{n}}$ and $W_{y_{n}}$ are $x_{n}=2Q_{n}+1$ and $y_{n}=2Q_{n}+2$, respectively. Thus, the transmitted signal of the proposed system can be expressed as

(1) $$e\left(t\right)=c_{R}\left(t\right)\cos\left(2\pi f_{0}t\right)+\sum_{n=1}^{N}m_{n}(t)\cos\left(2\pi f_{n}t\right)\;$$

where

(2) $$c_{R}\left(t\right)=\sum_{k=1}^{\beta }U_{k}h\left(t-kT_{c}\right)$$

and

(3) $$m_{n}\left(t\right)=\sum_{k=1}^{\beta }V_{n,k}h\left(t-kT_{c}\right)$$

are, respectively, the reference signal and M-ary information-bearing signal. $W_{R}$ is another Walsh code sequence which is utilized for processing the reference sequence, and ${U=W}_{R}⊗c$. $W_{R}$ is different from the paired Walsh codes sequences $W_{x_{n}}$ and $W_{y_{n}}$. $V_{n}={a_{n}W}_{x_{n}}⊗c+b_{n}W_{y_{n}}⊗c$. ⊗ is the Kronecker product. The length of the Walsh code sequence is P. Because the number of the index bits is $p_{1}$, the maximum value of $Q_{n}$ is $2^{p_{1}}$. Therefore, the length of the Walsh code sequence must meet $P\geq 2\cdot 2^{p_{1}}+2$. $T_{c}$ is the chip duration of discrete-time chaotic signal. $a_{n}$ and $b_{n}$ are, respectively, the in-phase and quadrature components of the $n^{th}$ M-ary constellation symbol $S_{n}=a_{n}+jb_{n}$. h(t) is the pulse response function of a square-root-raise-cosine filter. $f_{0},\;f_{1},f_{2}{\cdots f}_{N}$ are the frequencies of the subcarriers.

### 2.2. Receiver

**Figure 2:**
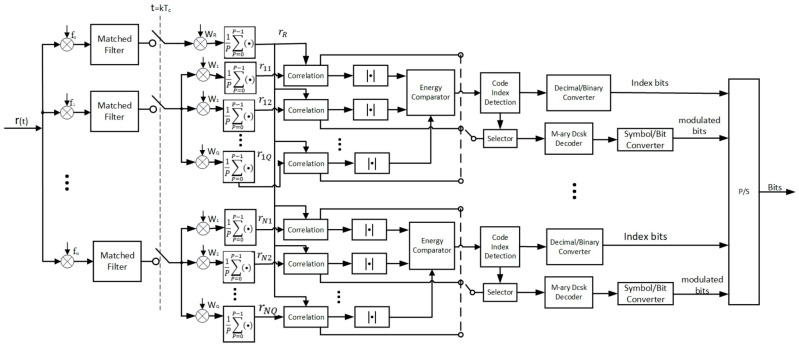
The block diagram of a MC-MCIM-MDCSK receiver.

Figure 2 shows the block diagram of a MC-MCIM-MDCSK receiver. The transmitted signal is passed through the multipath fading channel. The received signal can be obtained as

(4) $$r\left(t\right)=\sum_{l=1}^{L}\lambda_{l}e(t-\tau_{l})+\eta \left(t\right)$$

where L is the number of the propagation path. $\lambda_{l}$ is the channel coefficient of the $l^{th}$ path. $\tau_{l}$ is the path delay of the $l^{th}$ path. η(t) is the AWGN with zero mean and variance of $\frac{N_{0}}{2}$.

In Figure 2, the received signal $r\left(t\right)$ is processed by different subcarriers and matched filters. The results of the matched filters are sampled at the time instant $kT_{c},k=1,2,\cdots ,\beta$. The averaged reference signal is obtained as

(5) $$r_{R,j}=\frac{1}{P}\sum_{p=0}^{P-1}W_{R,p+1}\left[\sum_{l=1}^{L}\lambda_{l}W_{R,p+1}c_{P\theta +j-\tau_{l}}+\eta_{P\theta +j}\right]=\sum_{l=1}^{L}\lambda_{l}c_{j-\tau_{l}}+\eta_{R,j}\;$$

where

(6) $$\eta_{R,j}=\frac{1}{P}\sum_{p=0}^{P-1}W_{R,p+1}\cdot \eta_{P\theta +j}\;$$

The average information-bearing signal of the $n^{th}$ group is obtained as

(7) $$\begin{array}{c} r_{nq,j}=\frac{1}{P}\sum_{p=0}^{P-1}W_{nq,p+1}[\sum_{l=1}^{L}\lambda_{l}\left(a_{n}W_{x_{n},p+1}c_{P\theta +j-\tau_{l}}+b_{n}W_{y_{n},p+1}c_{P\theta +j-\tau_{l}}\right)+\eta_{P\theta +j}]\\ \;\;=\frac{1}{P}\sum_{p=0}^{P-1}W_{nq,p+1}\cdot \sum_{l=1}^{L}\lambda_{l}a_{n}W_{x_{n},p+1}c_{P\theta +j-\tau_{l}}\\ \;\;\;+\frac{1}{P}\sum_{p=0}^{P-1}W_{nq,p+1}\cdot \sum_{l=1}^{L}\lambda_{l}b_{n}W_{y_{n},p+1}c_{P\theta +j-\tau_{l}}+\frac{1}{P}\sum_{p=0}^{P-1}W_{nq,p+1}\cdot \eta_{P\theta +j}\\ \;\;\;\;\;\;\;\;\;=\left\{\begin{array}{c} \sum_{l=1}^{L}\lambda_{l}a_{n}c_{j-\tau_{l}}+\eta_{x,j},\;q=x_{n}\\ \sum_{l=1}^{L}\lambda_{l}b_{n}c_{j-\tau_{l}}+\eta_{y,j},\;q=y_{n}\\ \eta_{q,j},\;q\neq x_{n}\;and\;q\neq y_{n} \end{array}\right. \end{array}$$

where

(8) $$\eta_{x,j}=\frac{1}{P}\sum_{p=0}^{P-1}W_{nq,p+1}\cdot \eta_{P\theta +j}$$

(9) $$\eta_{y,j}=\frac{1}{P}\sum_{p=0}^{P-1}W_{nq,p+1}\cdot \eta_{P\theta +j}$$

(10) $$\eta_{q,j}=\frac{1}{P}\sum_{p=0}^{P-1}W_{nq,p+1}\cdot \eta_{P\theta +j},q\neq x_{n}\;and\;q\neq y_{n\;}$$

The output of the correlation can be obtained as

(11) $$\begin{array}{c} I_{nq,j}=\sum_{j=1}^{\theta }r_{R,j}\cdot r_{nq,j}\\ =\left\{\begin{array}{c} \sum_{l=1}^{L}\lambda_{l}^{2}a_{n}c_{j-\tau_{l}}^{2}+\sum_{j=1}^{\theta }\left[\sum_{l=1}^{L}\lambda_{l}c_{j-\tau_{l}}\eta_{x,j}+\sum_{l=1}^{L}\lambda_{l}a_{n}c_{j-\tau_{l}}\eta_{R,j}+\eta_{R,j}\cdot \eta_{x,j}\right],\;q=x_{n}\\ \sum_{l=1}^{L}\lambda_{l}^{2}b_{n}c_{j-\tau_{l}}^{2}+\sum_{j=1}^{\theta }\left[\sum_{l=1}^{L}\lambda_{l}c_{j-\tau_{l}}\eta_{y,j}+\sum_{l=1}^{L}\lambda_{l}b_{n}c_{j-\tau_{l}}\eta_{R,j}+\eta_{R,j}\cdot \eta_{y,j}\right],\;q=y_{n}\\ \sum_{j=1}^{\theta }\left[\sum_{l=1}^{L}\lambda_{l}c_{j-\tau_{l}}+\eta_{R,j}\right]\cdot \eta_{q,j}=\sum_{j=1}^{\theta }\sum_{l=1}^{L}\lambda_{l}c_{j-\tau_{l}}\eta_{q,j}+\sum_{j=1}^{\theta }{\eta_{R,j}\eta }_{q,j},q\neq x_{n}\;and\;q\neq y_{n} \end{array}\right. \end{array}$$

The energy comparation is performed on the decision variable $\left|I_{nq,j}\right|$. In order to estimate the index bits, the index of the maximum energy in each group is selected. The result of the energy comparation is utilized for obtaining the decimal value of the code index. The estimated value of the index bits can be obtained by the decimal-binary converter.

The M-ary constellation symbols corresponding to the indices of the maximum energy in each group can be estimated by using the Euclidean distance decision method. The modulated bits are then recovered by the symbol-to-bit converters. Finally, the data bits are recovered by the parallel-to-serial converter.

## 3. Performance Analysis

### 3.1. BER

Without loss of generality, it is assumed that the largest multipath delay is much shorter than the symbol duration. Thus, the intersymbol interference could be negligible [19,20]. We also assume that the channel is slowly fading, and the channel coefficients are constant during the one symbol.

Based on the Equation (11), the mean and the variance of the decision variable $I_{nq,j}$ are obtained as

(12) $$E[I_{nq,j}]=\left\{\begin{array}{c} \frac{1}{2}\sum_{l=1}^{L}\lambda_{l}^{2}\theta E\left[c_{j-\tau_{l}}^{2}\right]=\mu_{1},q=x_{n}\;or\;q=y_{n}\\ 0,\;q\neq x_{n}\;and\;q\neq y_{n} \end{array}\right.$$

(13) $$Var[I_{nq,j}]=\left\{\begin{array}{c} \frac{3}{4}\sum_{l=1}^{L}\lambda_{l}^{2}\theta \frac{N_{0}}{2}E\left[c_{j}^{2}\right]+\frac{\theta N_{0}^{2}}{4}=\sigma_{1}^{2},q=x_{n}\;or\;q=y_{n}\\ \sum_{l=1}^{L}\lambda_{l}^{2}\theta \frac{N_{0}}{2}E\left[c_{j}^{2}\right]+\frac{\theta N_{0}^{2}}{4}=\sigma_{2}^{2},\;q\neq x_{n}\;and\;q\neq y_{n} \end{array}\right.$$

Here, we set $a_{n}=b_{n}$.

Based on the cumulative distribution function (CDF) of the random variables, the symbol error rate (SER) of index bits can be obtained as [21,22]

(14) $$\begin{array}{c} P_{SER}=Prob[\left|I_{nx_{n},j}\right|<max(\left|I_{nq,j}\right|)\left|\;q\neq x_{n}\right.]\\ =1-\int_{0}^{+\infty }(F_{\left|I_{nq,j}\right|}(r){)}^{2^{p_{1}}-1}\cdot f_{\left|I_{nx_{n},j}\right|}(r)dr \end{array}$$

where

(15) $$f_{\left|I_{nx_{n},j}\right|}\left(r\right)=\frac{1}{\sqrt{2\pi \sigma_{1}^{2}}}[\exp\left(-\frac{{\left(r-\mu_{1}\right)}^{2}}{2\sigma_{1}^{2}}\right)+\exp\left(-\frac{{\left(r+\mu_{1}\right)}^{2}}{2\sigma_{1}^{2}}\right)]$$

(16) $$F_{\left|I_{nq,j}\right|}\left(r\right)=\mathrm{erf}\left(\frac{r}{\sqrt{2\sigma_{2}^{2}}}\right),\;q\neq x_{n}$$

The BER of the index bits can be calculated as

(17) $$\begin{array}{c} P_{ind}=\frac{2^{p_{1}-1}}{2^{p_{1}}-1}P_{SER}\\ =\frac{2^{p_{1}-1}}{2^{p_{1}}-1}\{1-\int_{0}^{+\infty }(\mathrm{erf}\left(\frac{r}{\sqrt{2\sigma_{1}^{2}}}\right){)}^{2^{p_{1}}-1}\cdot \frac{1}{\sqrt{2\pi \sigma_{1}^{2}}}[\exp\left(-\frac{{\left(r-\mu_{1}\right)}^{2}}{2\sigma_{1}^{2}}\right)+\exp\left(-\frac{{\left(r+\mu_{1}\right)}^{2}}{2\sigma_{1}^{2}}\right)]dr\} \end{array}$$

To calculate $P_{mod}$, we consider two cases. First, the index bits are recovered correctly but the modulated bits are incorrect. Second, the modulated bits are recovered correctly but the index bits are incorrect. Thus, the BER of the modulated bits $P_{mod}$ can be obtained as

(18) $$P_{mod}=P_{MDCSK}\left(1-P_{SER}\right)+\frac{M-1}{M\log_{2}M}P_{SER}$$

where

(19) $$P_{MDCSK}=\frac{2}{\log_{2}M}\int_{-\pi /M}^{\pi /M}(\frac{\exp(-\frac{\rho^{2}}{8})}{2\pi }+\exp(-\frac{\rho^{2}\sin^{2}\phi }{8})\cdot \frac{\rho \cos\frac{\phi }{2}}{\sqrt{2\pi }}\cdot Q(-\frac{\rho \cos\phi }{2}))d\phi$$

where $\rho =\frac{2\gamma_{s}}{\sqrt{2\gamma_{s}+\beta }}$, $Q\left(x\right)=\frac{1}{2\pi }\int_{x}^{\infty }\exp^{-\frac{t^{2}}{2}}dt$, $\gamma_{s}=\frac{E_{s}}{N_{0}}$. $E_{s}$ is the symbol energy.

Based on Equations (17) and (18), the BER of the proposed system with AWGN channel can be given as

(20) $$P_{system}=\frac{p_{1}}{p_{1}+p_{2}}P_{ind}+\frac{p_{2}}{p_{1}+p_{2}}P_{mod}$$

A L-path Rayleigh fading channel is considered. The probability density function (PDF) of the symbol-SNR $\gamma_{b}$ can be written as [23,24,25,26,27]

(21) $$f\left(\gamma_{b}\right)=\frac{\gamma_{b}^{L-1}}{{\left(L-1\right)!\bar{\gamma }}_{c}^{L}}exp(-\frac{\gamma_{b}}{{\bar{\gamma }}_{c}})$$

where the average bit SNR per channel is ${\bar{\gamma }}_{c}=\frac{E_{b}}{N_{0}}E[\lambda_{l}^{2}]$, and $\gamma_{b}=\frac{E_{b}}{N_{0}}\sum_{l=1}^{L}\lambda_{l}^{2}$ with $\sum_{l=1}^{L}{E[\lambda }_{l}^{2}]=1$. $E_{b}$ is the bit energy. Thus, the BER of the proposed system under multipath Rayleigh fading channel can be obtained by

(22) $$P_{multi-path}=\int_{0}^{+\infty }P_{system}f\left(\gamma_{b}\right)d\gamma_{b}$$

### 3.2. Efficiency Discussions

**Figure 3:**
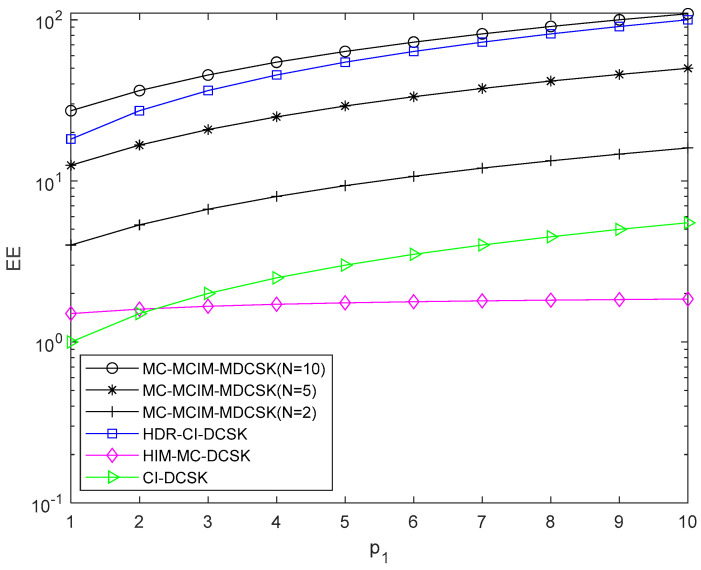
EE comparisons with the systems.

**Figure 4:**
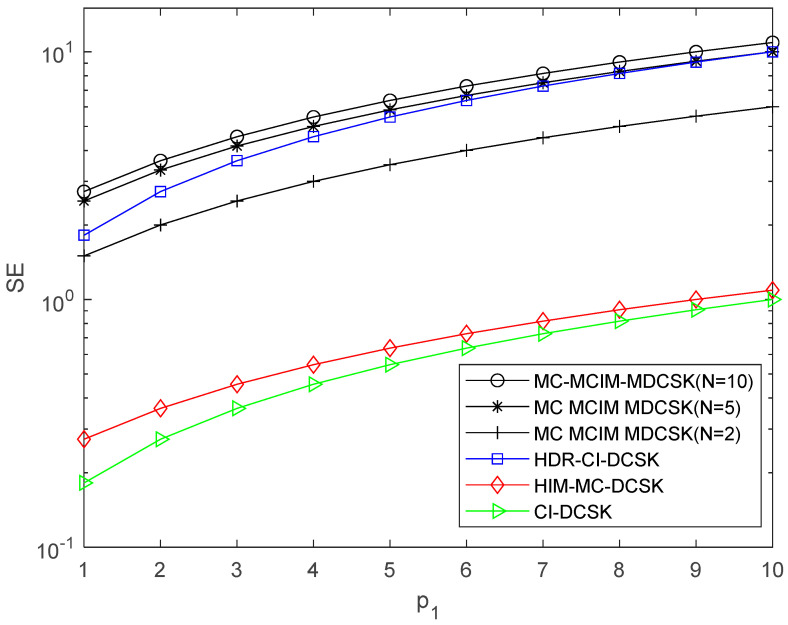
SE comparisons with the systems.

**Figure 5:**
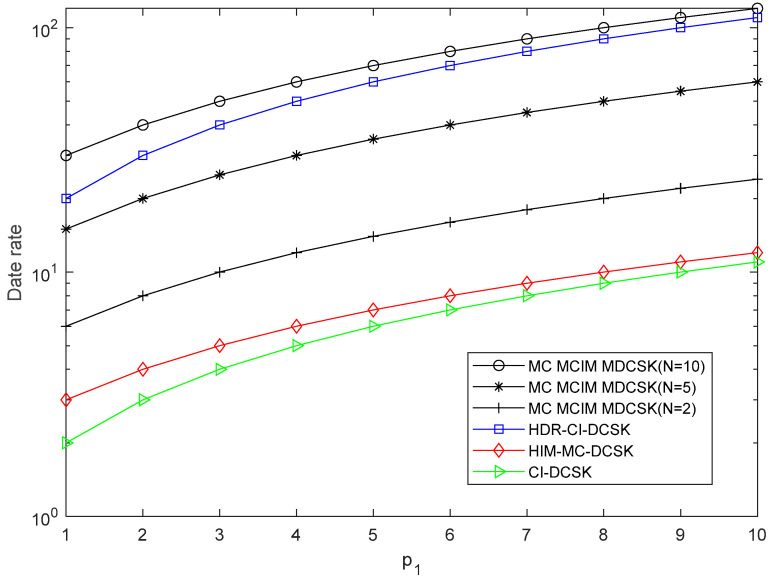
Data rate comparisons with the systems.

The comparisons of EE, SE, and the data rate are given in this section, which is shown in Table 1. N is the number of the subcarriers. For visualization, the EE, SE, and the data rate comparisons are also shown in Figure 3, Figure 4 and Figure 5. We set $p_{2}=2$. It can be observed that the proposed system performs the best in EE, SE, and the data rate when N=10.

To research the EE, we calculate the transmitted data-energy-to-bit-energy ratio (DBR) [10]:

(23) $$DBR=\frac{E_{data}}{E_{b}}\;$$

where $E_{data}$ is the energy to transmit the data, and $E_{b}$ is the transmitted bit energy.

The definition of SE is the number of bits transmitted per carrier in one symbol duration [28].

### 3.3. Complexity

The comparisons of the complexity using the Big-O method [29,30,31] are shown in Table 2. Here, we mainly consider time complexity and memory complexity. The analysis of time complexity includes the initialization of the proposed system parameters and chaotic sequence generation, the process of modulation, the process of noise adding, the process of demodulation, the calculation of BER, and so on. The total time complexity is $O(\delta \cdot (N\cdot \theta +2N\cdot P\cdot \theta +N(p_{1}+p_{2})P\cdot \theta ))$. δ represents the length of the signal-to-noise ratio value. Homoplastically, the total memory complexity is $O(N+N\left(p_{1}+p_{2}\right)+P^{2}+N(p_{1}+p_{2})P\cdot \theta +N\cdot P\cdot \theta +H\cdot \delta )$. H represents the number of runs. In Big-O analysis, we usually focus on the highest-order terms because when the input size is large, the lower-order and constant terms have a negligible effect on the overall complexity. Thus, the simplified time complexity is $O(\delta \cdot (\;N(p_{1}+p_{2})P\cdot \theta ))$, and the simplified memory complexity is $O(N(p_{1}+p_{2})P\cdot \theta$).

Obviously, the memory complexity and the time complexity of the proposed system are smaller than the HDR CI-DCSK system and the CI-DCSK system [32], but higher than the HIM-MC-DCSK system [33]. That is, the complexity of the proposed system is relatively moderate.

## 4. Simulation Results and Discussions

In this section, the BER performance of the proposed MC-MCIM-MDCSK system over the multipath Rayleigh fading channel and the AWGN channel is shown. In computer simulations, delays of the propagation paths in a three-path Rayleigh fading channel with equal power gains are τ_1_ = 0, τ_2_ = 2, and τ_3_ = 5. This three-path Rayleigh fading channel is usually adopted in many chaos-based communication systems [33,34,35]. The Monte Carlo method is used in the paper [33,36,37,38,39,40,41,42]. The greater the number of simulations, the closer the experimental data is to the theoretical data.

**Figure 6:**
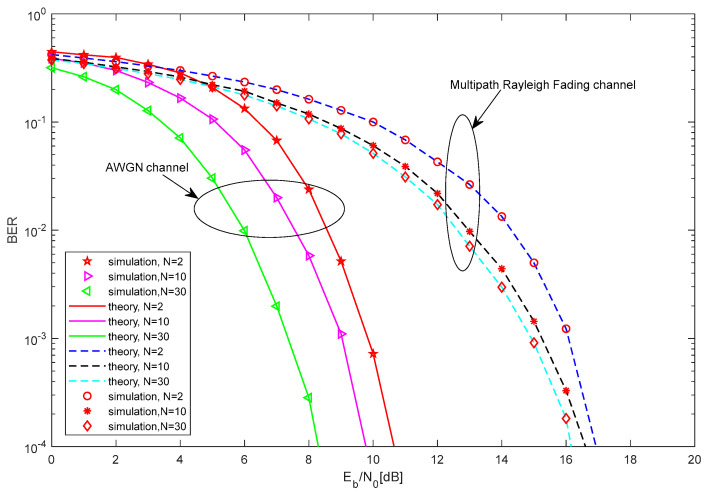
The BER performance comparison between the theoretical and the simulated results of the proposed MC-MCIM-MDCSK system.

**Figure 7:**
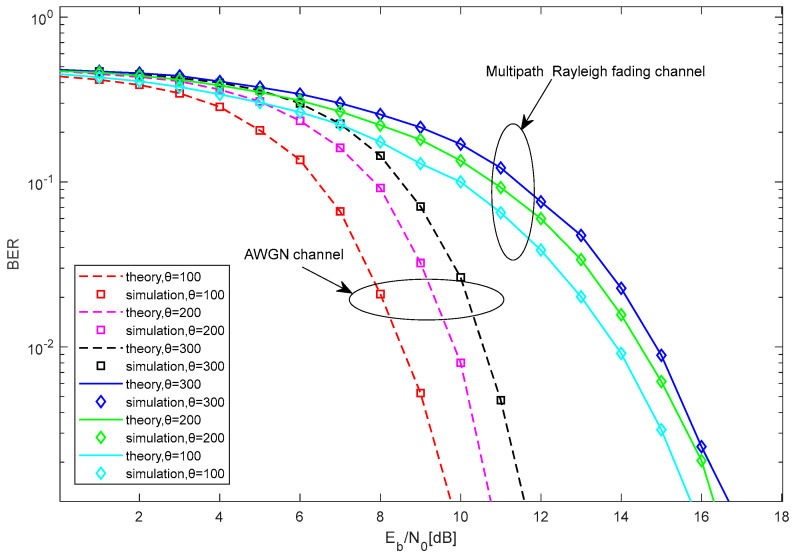
The BER performance comparison between the analytical and the simulated results of the proposed system.

The BER performance comparison between the theoretical and the simulated results of the proposed MC-MCIM-MDCSK system is shown in Figure 6 and Figure 7. Clearly, the simulation results are essentially consistent with the theoretical results. This further confirms the correctness and effectiveness of the theoretical analysis.

In Figure 6, the BER performance of the proposed system is shown with M=4, θ=20, and different N. Clearly, the BER performance of the proposed system improves when the number of the subcarriers increases. In fact, the length of the Walsh codes will increase when the number of the subcarriers increases. And the noise variance will decrease when the length of the Walsh codes increases. Thus, the BER performance improves when the number of the subcarriers increases.

In Figure 7, the BER performance of the proposed system is shown with M=4, N=10, P=16, and different θ. Clearly, when the length of chaotic signal decreases, the BER performance of the proposed system improves. It is because the noise component in noise-noise correlation term will become more significant when the length of chaotic signal increases, which will result in a relatively poor BER performance.

**Figure 8:**
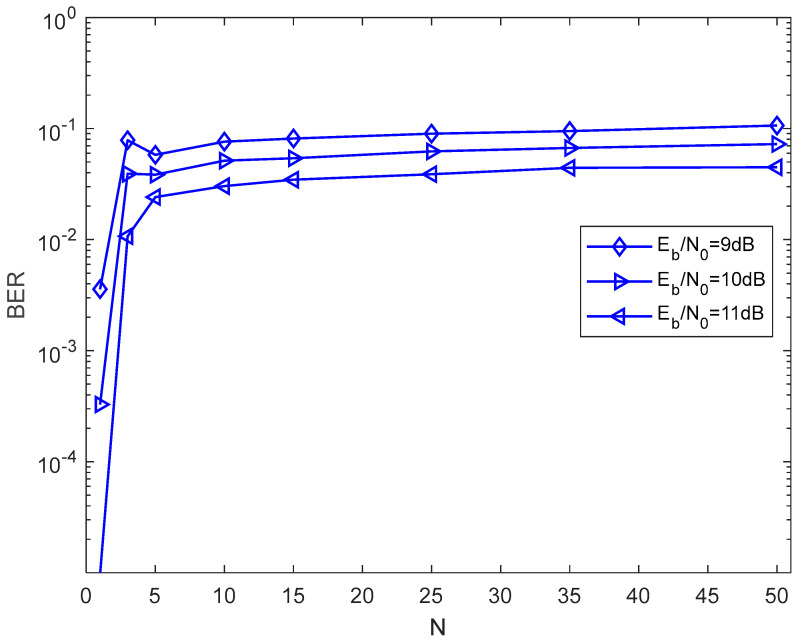
BER performance of the proposed system versus the number of subcarriers over an AWGN channel with θ=100, P=16, M=4.

In Figure 8, the effect of a different number of subcarriers on the BER of the proposed system over the AWGN channel is shown. Clearly, when the length of the chaotic signal, the length of the Walsh codes, and the $\frac{E_{b}}{N_{0}}$ are fixed, the BER performance of the proposed system improves as the number of subcarriers increases. It is observed that the BER performance improves greatly when N increases from 1 to 15. When N increases from 16 to 50, the BER performance improves slowly.

**Figure 9:**
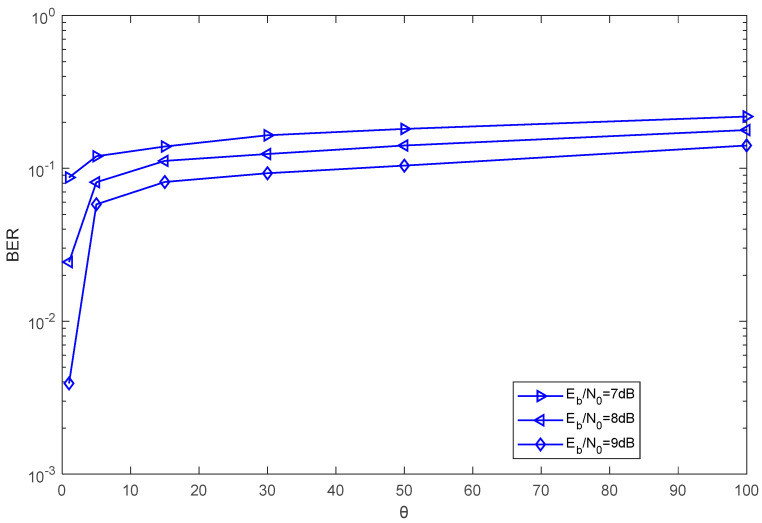
The BER performance of the proposed system versus the length of the chaotic signal over an AWGN channel with M=4, N=10, P=16.

In Figure 9, the effect of the different lengths of the chaotic signal on the BER of the proposed system over the AWGN channel is shown. Clearly, when the number of subcarriers, the length of the Walsh codes, and the $\frac{E_{b}}{N_{0}}$ are fixed, the BER performance of the proposed system improves as the length of the chaotic signal decreases. The curve trend of the BER performance is the same as that of the BER curves in Figure 7.

**Figure 10:**
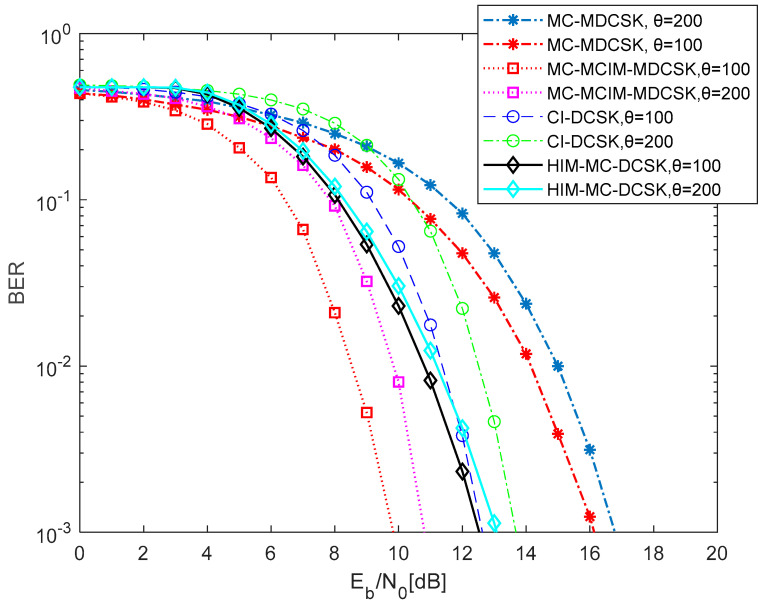
The comparison of the BER performance between the proposed system and the existing system with different θ.

In Figure 10, the comparison of the BER performance between the proposed system and the existing system over the AWGN channel is shown. Here the existing system includes the MC-MDCSK system [35], the CI-DCSK system [32] and the HIM-MC-DCSK system [33]. The number of the parallel group is set to N=10, the length of the Walsh code is set to P=16, and the scale is set to M=16. Obviously, the BER performance of the proposed system is better than the MC-MDCSK system, the CI-DCSK system, and the HIM MC-DCSK system when some parameter settings are the same. For instance, when θ=100, the gain of the proposed MC-MCIM-MDCSK system is about 6 dB at a BER of $10^{-3}$ over the AWGN channel.

**Figure 11:**
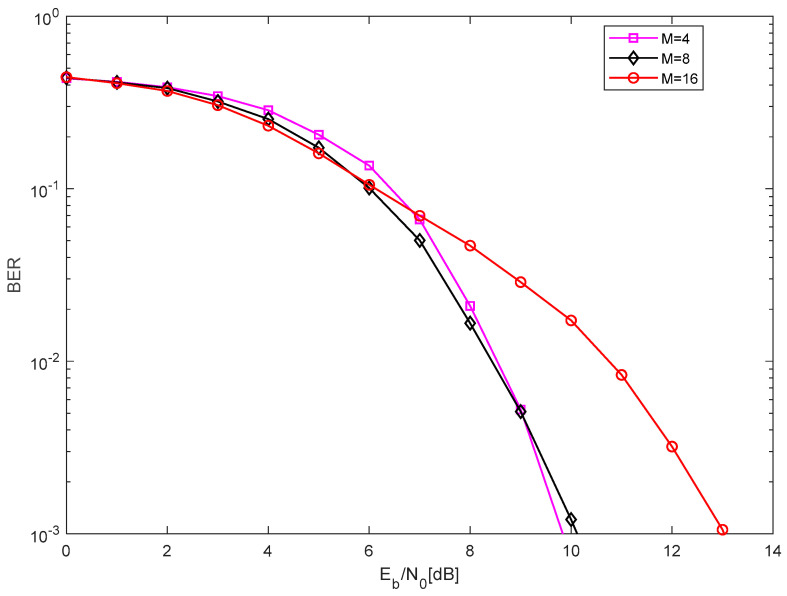
The comparison of the BER performance with different *M* over the AWGN channel.

In Figure 11, the comparison of the BER performance with different M over the AWGN channel is shown. The length of the chaotic signal is set to θ=100, the length of the Walsh code is set to P=64, and the number of the parallel group is set to N=10. Clearly, the BER performance is almost relative when *M* = 4, 8, and better than the BER performance when M=16.

**Figure 12:**
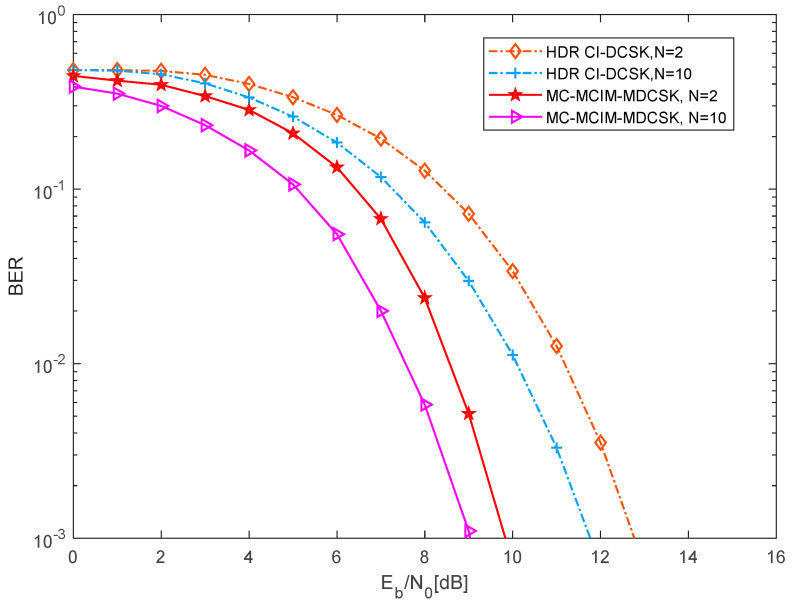
The comparison of the BER performance between the proposed system and the HDR CI-DCSK system with different N.

In Figure 12, the comparison of the BER performance between the proposed system and the HDR CI-DCSK system [21] over the AWGN channel is shown. The length of the Walsh code is set to P=16. The length of the chaotic signal is set to θ=20, and the scale is set to M=4. Obviously, the BER performance of the proposed system is better than the HDR CI-DCSK system when some parameter settings are the same. For instance, when N=2, the gain of the proposed MC-MCIM-MDCSK system is about 3 dB at a BER of $10^{-3}$ over the AWGN channel.

## 5. Conclusions

In this paper, the modified code index modulation scheme based on a multi-carrier M-ary DCSK system has been proposed, referred to as a MC-MCIM-MDCSK system. In the proposed system, the modified code index modulation scheme is used, which selects two Walsh codes for realizing M-ary modulation. The serial data are divided N parallel groups and each group includes index bits and modulated bits. The reference chaotic signal and N parallel groups are transmitted by N+1 subcarriers. The EE, SE, and the data rate of the proposed system are compared with other systems. When the number of parallel groups is relatively large, the proposed system has better EE, SE, and a high data rate in comparison with its competitors. The newly proposed system combines multi-carrier and code indexing techniques to simultaneously improve the EE, SE, and data rate of the system, as well as the BER performance. Multiple parallel signals carry information on mutually orthogonal Walsh codes. The complexity of the proposed system is lower than that of the CI-DCSK system and the HDR CI-DCSK system, but higher than that of the HIM-MC-DCSK system. The theoretical BER performance expressions are obtained over AWGN and the multipath Rayleigh fading channels, which are consistent with the simulation results. Simulation results show that the BER performance will improve when the length of the chaotic signal decreases and the number of the parallel groups increases. The BER performance of the proposed system runs better with M=4,8 than M=16. The comparison between the proposed system and other systems shows that the proposed system has a better BER performance than its competitors. Because of the excellent performance, the proposed system will be an outstanding candidate for communication applications.

## Figures and Tables

**Table 1:** Comparisons between systems.

**Performance**	**MC-MCIM-MDCSK**	HDR CI-DCSK	HIM-MC-DCSK	CI-DCSK
EE	$\frac{N^{2}(p_{1}+p_{2})}{N+1}$	$\frac{N^{2}(p_{1}+1)}{N+1}$	$\frac{2N}{N+1}$	$\frac{p_{1}+1}{2}$
SE	$\frac{N(p_{1}+p_{2})}{N+1}$	$\frac{N(p_{1}+1)}{N+1}$	$\frac{p_{1}+p_{2}}{N+1}$	$\frac{p_{1}+1}{N+1}$
Data rate	$N(p_{1}+p_{2})$	$N(p_{1}+1)$	$p_{1}+p_{2}$	$p_{1}+1$

**Table 2:** The comparisons of the complexity.

**Complexity**	**MC-MCIM-MDCSK**	HDR CI-DCSK	HIM-MC-DCSK	CI-DCSK
Time complexity	$O(H\cdot \delta \cdot (\;N(p_{1}+p_{2})P\cdot \theta ))$	$O(H\cdot \delta \cdot N^{2}\cdot 2^{p_{1}}\cdot P\cdot \theta )$	$O(H\cdot \delta \cdot N\cdot p_{1}\cdot \theta )$	$O(H\cdot (\delta +1){\cdot 2}^{p_{1}}\cdot \;\theta )$
Memeory complexity	$O(N(p_{1}+p_{2})P\cdot \theta$)	$O((1+N\cdot 2^{p_{1}})\cdot P\cdot \theta )$	$O((N\cdot p_{1}+1)\cdot \theta )$	O( $\left(2^{p_{1}}+1)\;\cdot \;\theta \right)$

## Data Availability

Data is contained within the article.
